# From *Rerum Novarum* to *Magnifica Humanitas*: Catholic Social Doctrine and Decent Work

**DOI:** 10.5334/aogh.5366

**Published:** 2026-07-16

**Authors:** Raimondo Leone, Paolo Nicosia, Eugenio Cunego, Walter Ricciardi

**Affiliations:** 1 Department of Life Sciences and Public Health, Università Cattolica del Sacro Cuore, Largo Francesco Vito 1, 00168 Rome, Italy; 2 Pontifical Lateran University, Rome, Italy; 3 Department of Life Sciences, Health and Healthcare Professions, Link Campus University, Rome, Italy

**Keywords:** Catholic social teaching, *Rerum Novarum*, *Dilexi Te*, *Magnifica Humanitas*, decent work, occupational medicine, occupational health, workers’ rights, Pope Leo XIII, Pope Leo XIV, artificial intelligence, ILO

## Abstract

The Catholic Church has developed a rich body of social teaching addressing the dignity of work and the protection of workers’ health since the late nineteenth century. This article examines the trajectory of Catholic Social Teaching (CST) from Pope Leo XIII’s encyclical *Rerum Novarum* (1891) to Pope Leo XIV’s recent magisterium, including the apostolic exhortation *Dilexi Te* (2025) and the encyclical *Magnifica Humanitas* (2026) on safeguarding the human person in the time of artificial intelligence, with a particular focus on the concept of decent work as articulated by the International Labour Organization (ILO) and its intersection with Occupational Medicine (OM). The analysis highlights that Catholic social thought has consistently advocated for working conditions that safeguard the physical, mental, and social well-being of workers, anticipating many principles later codified in international labor standards. It further contends that *Magnifica Humanitas* extends this tradition to the challenges posed by digital technology and artificial intelligence to the dignity of work. The article argues that the convergence between CST and modern occupational health frameworks provides a compelling ethical foundation for advancing decent work and Occupational Health Services (OHS) worldwide.

## Introduction

The relationship between work, human dignity, and health has been a central concern of moral philosophy and social ethics for centuries. Within the Catholic tradition, this concern found its most systematic expression in what is now known as Catholic Social Teaching (CST), a body of teaching that addresses the social, economic, and political dimensions of human life in light of the Gospel and natural law [1]. CST has consistently affirmed that human work, even in its most ordinary and demanding physical manifestations, is not merely an economic activity but a fundamental expression of human personhood, carrying with it inherent rights and responsibilities that transcend market dynamics [2, 3]. As Sison and Fontrodona have argued, the Catholic tradition provides a distinctive framework for understanding the dignity of work, one rooted in a vision of the common good that integrates personal fulfillment with social responsibility [4]. At the international level, the concept of decent work, as formulated by the International Labour Organization (ILO), represents a parallel effort to establish normative standards for the protection and promotion of workers’ well-being. The ILO defines decent work as productive employment carried out in conditions of freedom, equity, security, and human dignity [5]. Central to this framework is the role of Occupational Medicine (OM) and Occupational Health Services (OHS), which aim to promote and maintain the highest degree of physical, mental, and social well-being of workers in all occupations [6]. The Decent Work Agenda (DWA) has since been reinforced by its inclusion in the United Nations (UN) 2030 Agenda for Sustainable Development, specifically under Sustainable Development Goal (SDG) 8, which calls for the promotion of sustained, inclusive, and sustainable economic growth, full and productive employment, and decent work for all [7].

This article traces the development of CST’s teaching on work and workers’ health from Pope Leo XIII’s landmark encyclical *Rerum Novarum* (1891) to Pope Leo XIV’s apostolic exhortation *Dilexi Te* (2025) and his first encyclical *Magnifica Humanitas* (2026), examining the points of convergence between Catholic social thought and the modern frameworks of decent work and occupational health. By doing so, it seeks to demonstrate that these two traditions, although arising from distinct sources, theological reflection on the one hand and international labor governance on the other, share a profound commitment to the integral protection of the working person.

## Historical Foundations: *Rerum Novarum* and the Birth of Catholic Social Teaching

### The social question and the industrial revolution

The second half of the nineteenth century witnessed an unprecedented transformation in the nature of work. The Industrial Revolution brought about mass urbanization, the concentration of workers in factories, and the emergence of labor conditions that were often dangerous, exhausting, and dehumanizing [8]. Workers, including women and children, faced extremely long working hours, exposure to hazardous substances, inadequate ventilation, and a near-total absence of safety measures. Occupational injuries and diseases were widespread, yet legal protections were minimal and medical attention for work-related ailments was virtually non-existent [9, 10]. It was in this context that Pope Leo XIII issued *Rerum Novarum* (“Of New Things”) on May 15, 1891, the first papal encyclical to address directly the social and economic conditions of the working class [11]. The encyclical is widely regarded as the founding document of modern CST, establishing the Church’s moral authority to speak on matters of labor, wages, and the organization of economic life [12, 13]. As Curran has noted, *Rerum Novarum* inaugurated a tradition of sustained papal engagement with social questions that would unfold over more than a century [12].

### Key principles of *Rerum Novarum* relevant to workers’ health

*Rerum Novarum* articulated several principles that bear directly on the protection of workers’ physical and mental health. First, the encyclical affirmed the inherent dignity of work, grounding it in the example of Christ himself, who spent much of his earthly life as a carpenter [11]. This theological anthropology implies that work must never be reduced to a mere commodity; rather, it must be organized in a manner that respects the worker as a person [4, 14]. Second, Pope Leo XIII explicitly condemned the exploitation of workers through excessive labor. He stated that employers must not treat human beings as instruments for profit, emphasizing that it is unjust to impose working conditions that degrade the mind and exhaust the body [11]. Third, the encyclical recognized the right of workers to reasonable working hours, to legitimate rest, and to conditions that do not endanger health. It held that the State has a duty to intervene to protect the defenseless and the poor when market forces fail to do so [12, 13]. Fourth, *Rerum Novarum* upheld the right to a just wage sufficient to support the worker and his family, acknowledging the connection between economic security and overall well-being. In the United States (US), Father John Ryan’s *A Living Wage* (1906) drew directly upon this encyclical to argue that a living wage is a natural right grounded in the intrinsic worth of the human person [15]. Fifth, the encyclical affirmed the right of workers to form associations and unions, recognizing collective organization as indispensable for the defense of labor rights [11, 16]. These principles, although articulated in a pre-modern medical framework, anticipated many of the concerns that would later become central to occupational health policy.

## The Development of Catholic Social Teaching on Work and Health

### From *Quadragesimo Anno* to *Laborem Exercens*

The principles established by *Rerum Novarum* were subsequently developed and enriched by successive papal documents. Pope Pius XI’s encyclical *Quadragesimo Anno* (1931), issued on the 40th anniversary of *Rerum Novarum*, reaffirmed the rights of workers and expanded the analysis of structural injustice in economic systems [17]. Pope John XXIII’s *Mater et Magistra* (1961) broadened the scope of CST to include global development and the obligation of wealthier nations toward poorer ones [18], while Pope Paul VI’s *Populorum Progressio* (1967) further developed the concept of integral human development, asserting that authentic development must encompass the whole person and every person [19]. Of particular significance for the theme of workers’ health is Pope John Paul II’s encyclical *Laborem Exercens* (1981), issued on the 90th anniversary of *Rerum Novarum*. In this document, Pope John Paul II developed a comprehensive theology of work, arguing that the value of human labor derives not from the type of work performed but from the fact that the person performing it is endowed with dignity [20]. He distinguished between the objective and subjective dimensions of work, establishing the priority of the latter; a principle that Finn has identified as a decisive contribution to the Catholic understanding of economic life [21]. He also stressed the importance of labor unions and the right to strike, and he addressed the need for working conditions to be adapted to the capabilities and needs of the individual worker, a principle that resonates with the modern concept of occupational health surveillance [20].

### *Centesimus Annus* and *Caritas in Veritate*: Toward integral human development

Pope John Paul II’s *Centesimus Annus* (1991), commemorating the centenary of *Rerum Novarum*, argued that integral human development in the workplace is not opposed to productivity and efficiency but rather enhances them [22]. This insight has profound implications for occupational health, suggesting that investment in workers’ well-being is not merely a moral imperative but also a sound economic strategy, a view increasingly supported by empirical evidence in the occupational health literature [23, 24]. It also still asks for a deeper reflection about the very notion of human work and its priority with respect to any economic consideration and commitment, as it is strictly related to the notion of human dignity. Pope Benedict XVI’s *Caritas in Veritate* (2009) made what is perhaps the most explicit connection between CST and the concept of decent work. Pope Benedict XVI outlined seven defining principles of what constitutes decent work: it must express the essential dignity of the person; it must be freely chosen; it must enable respect and freedom from discrimination; it must allow families to meet their needs; it must permit the free organization of workers; it must leave room for personal, familial, and spiritual development; and it must guarantee retirees a decent standard of living [25]. These criteria closely mirror the pillars of the ILO DWA, demonstrating a remarkable convergence between Catholic social thought and international labor standards [7, 25]. Yet the encyclical’s contribution extends beyond this convergence. At its core, *Caritas in Veritate* is structured around the concept of integral human development, a notion first articulated by Pope Paul VI in *Populorum Progressio* (1967) and substantially deepened by Pope Benedict XVI [19, 25]. Integral human development holds that authentic development cannot be reduced to economic growth or material well-being alone; it must encompass the moral, spiritual, relational, and social dimensions of the human person in their entirety. In this framework, decent work is not merely an instrument of economic participation but a constitutive element of human flourishing, a means by which persons realize their vocation to contribute to the common good and to grow in freedom and dignity [25]. Crucially, Pope Benedict XVI insists that this integral vision requires attention to the whole person and to the whole of humanity: no authentic development can occur when workers are reduced to factors of production, when their physical or mental health is compromised by unjust conditions, or when entire populations are excluded from the benefits of economic progress. This anthropological vision provides the conceptual bridge between the Church’s concern for the conditions of labor and its broader commitment to ecological responsibility: if development must be integral (encompassing every dimension of human life and every human being), it cannot be separated from the care of the natural environment that sustains all life. It is precisely this insight that Pope Francis would develop into the concept of integral ecology in *Laudato Si’*, extending the social doctrine of the Church into the environmental domain and establishing a unified framework within which the protection of workers and the protection of creation are understood as inseparable dimensions of a single moral imperative [26].

### *Laudato Si’* and *Fratelli Tutti*: Work, ecology, and fraternity

Pope Francis’s encyclical *Laudato Si’* (2015) introduced the concept of integral ecology, arguing that the environmental and social crises are inseparable dimensions of a single complex crisis [26]. The encyclical addressed the conditions of work explicitly, noting how technological innovation, social exclusion, and environmental degradation converge to threaten the dignity of workers, particularly in the Global South [26, 27]. Pope Francis warned that the dominant technocratic paradigm risks reducing the worker to a mere appendage of the machine, echoing the concerns first articulated by Pope Leo XIII [11, 26]. In *Fratelli Tutti* (2020), Pope Francis further developed the theme of universal fraternity, calling for an economic model that places the person at the center and that ensures decent working conditions for all, including migrants and those in the informal economy [28]. Both documents reinforced the conviction, already present in *Rerum Novarum*, that the protection of workers cannot be left to market forces alone but requires deliberate social, political, and institutional action [1, 28].

## *Dilexi Te* and *Magnifica Humanitas*: Pope Leo XIV and the Renewal of the Church’s Social Commitment

### Context and significance of Dilexi Te

On October 4, 2025, the feast of Saint Francis of Assisi, Pope Leo XIV signed his first apostolic exhortation, *Dilexi Te* (“I Have Loved You”), completing a document that had been substantially prepared by the late Pope Francis before his death on April 21, 2025 [29]. The exhortation builds upon the themes of Pope Francis’s final encyclical, *Dilexit Nos* (2024), on the devotion to the Sacred Heart of Jesus, extending its reflections to the Church’s commitment to the poor and the marginalized [30]. *Dilexi Te* comprises 121 paragraphs distributed across five chapters. Its fourth chapter (paragraphs 82–102) explicitly situates the contemporary concern for poverty within more than 130 years of CST, from *Rerum Novarum* to *Caritas in Veritate*, recalling the vision of a “Church of the poor” embraced by the Second Vatican Council and the teachings of the Latin American episcopal conferences of Medellín, Puebla, Santo Domingo, and Aparecida [29, 31]. Poverty and inequality are described as “structures of sin” reinforced by economic systems that exclude the weak, and the exhortation calls for the dismantling of such structures through the combined force of moral commitment, science, technology, and effective social policies [29, 32].

### *Dilexi Te* and the conditions of labor

Although *Dilexi Te* is primarily focused on love for the poor, its analysis of the structural causes of poverty has direct implications for the conditions of labor and, by extension, for occupational health. The exhortation rejects the notion that poverty is merely a matter of individual choice or fate, identifying instead the systemic economic forces that perpetuate exploitation and deprivation [29]. It warns against the assumption that free market mechanisms will automatically resolve the problem of poverty, calling instead for deliberate intervention grounded in justice and solidarity [32]. Pope Leo XIV, who spent many years as an Augustinian missionary in Peru, brings a particular sensitivity to the realities of labor in the Global South, where workers often endure hazardous conditions, inadequate wages, and limited access to healthcare and social protection [29–31]. The exhortation’s emphasis on the preferential option for the poor (a cornerstone of CST [1]) reinforces the moral imperative to ensure that all workers, especially the most vulnerable, have access to safe and healthy working environments [29]. Furthermore, in his early pronouncements, Pope Leo XIV has drawn attention to the challenges posed by artificial intelligence (AI) and technological disruption to human dignity, justice, and labor, calling for a response rooted in the Church’s social teaching [33, 34]. This concern extends the historical trajectory of CST from the first Industrial Revolution addressed by Pope Leo XIII to the contemporary technological transformation, underscoring the enduring relevance of the Church’s advocacy for the protection of workers.

### *Magnifica Humanitas*: The dignity of work in the age of artificial intelligence

If *Dilexi Te* situates the conditions of labor within the Church’s concern for the poor, Pope Leo XIV’s first encyclical, *Magnifica Humanitas* (2026), addresses head-on the transformation of work brought about by digital technology and AI [35]. Signed on May 15, 2026, the 135th anniversary of *Rerum Novarum*, the encyclical explicitly positions itself within the trajectory of social teaching inaugurated by Pope Leo XIII, presenting AI as the *res novae* of the present era, just as the conflict between capital and labor constituted the “new things” of the industrial age [35]. Subtitled “On Safeguarding the Human Person in the Time of Artificial Intelligence,” the document warns against a technocratic paradigm that risks reducing the human person to a data point, a labor unit, or an instrument of control, and it insists that the value of the person can never be measured by cognitive performance or economic productivity [35]. This anthropological claim stands in direct continuity with the subjective dimension of work articulated by Pope John Paul II in *Laborem Exercens* and with the integral human development at the heart of *Caritas in Veritate* [20, 25, 35].

The fourth chapter of the encyclical, devoted to safeguarding truth, work, and freedom, contains a dedicated treatment of “the dignity of work at a time of digital transition,” reaffirming the primacy of human labor over finance and productivity and addressing the threat that automation poses to employment security [35]. For the purposes of occupational health, this analysis is significant in at least three respects. First, the encyclical reframes the technocratic reduction of the worker not merely as an economic problem but as an anthropological one, echoing the concern, already raised by Pope Leo XIII and Pope Francis, that the worker must never become a mere appendage of the machine [11, 26, 35]. Second, by calling for regulatory tools and shared responsibility among scientists, entrepreneurs, workers, educators, and legislators, it provides a normative framework congruent with the ILO’s emphasis on social dialogue and governance [7, 35]. Third, its insistence that social justice must shape the very design of digital systems, rather than be retrofitted after deployment, anticipates a preventive logic familiar to OM, in which hazards are best addressed at the source rather than mitigated after harm has occurred [35, 36]. In this light, *Magnifica Humanitas* may be read as extending the historical arc of CST from the first Industrial Revolution to the algorithmic transformation of work, furnishing a renewed ethical foundation for the protection of workers’ physical, mental, and social well-being in an era of rapid technological change [35]. Its concluding appeal to remain “profoundly human” in the age of AI resonates directly with the convergence between Catholic social thought and the DWA explored in the sections that follow [35].

## Decent Work and Occupational Medicine: Bridging Ethical Principles and Health Practice

### The ILO decent work agenda

The concept of decent work was formally introduced by the ILO in 1999 under the leadership of Director-General Juan Somavia, as a comprehensive framework for achieving productive employment in conditions of freedom, equity, security, and human dignity [5]. The DWA rests on four strategic pillars: employment creation and enterprise development; social protection; standards and rights at work; and governance and social dialogue [7]. Each of these pillars intersects with the concerns of occupational health, as the protection of workers from occupational hazards and the promotion of workplace well-being are integral components of social protection and labor standards [37]. The DWA has been operationalized through the Decent Work Country Programmes (DWCP), which have been implemented in approximately two-thirds of the ILO’s Member States [7]. However, evaluations have revealed that occupational health components are often insufficiently developed within these programs, particularly in low- and middle-income countries (LMIC) where the burden of work-related diseases is disproportionately high [7, 38]. As Schulte et al. have argued, a more comprehensive framework is needed to integrate Occupational Safety and Health (OSH) perspectives into the construction of policies and practices supportive of decent work [39]. The measurement and operationalization of decent work have been the subject of considerable scholarly attention. Anker et al. developed a foundational framework identifying 11 substantive dimensions of decent work, encompassing employment opportunities, unacceptable work, adequate earnings, decent working time, stability and security, balancing work and family, fair treatment, safe work environment, social protection, social dialogue, and the economic and social context [40]. Bescond et al. subsequently proposed seven statistical indicators for international comparison, enabling cross-country assessments of decent work deficits [41]. The ILO consolidated these efforts in its manual on Decent Work Indicators (2012), providing standardized concepts and definitions for monitoring progress across Member States [42]. The inclusion of decent work in the United Nations Sustainable Development Goals as SDG 8, promoting sustained, inclusive, and sustainable economic growth, full and productive employment, and decent work for all, further elevated the concept to a central position in the global development agenda [43]. From a psychological perspective, the concept of decent work has been enriched by the Psychology of Working Theory (PWT) developed by Duffy, Blustein, and colleagues, which places decent work as the central construct linking social and economic constraints to individual well-being outcomes [44]. The PWT posits that contextual factors, including marginalization, economic conditions, and access to social capital, shape individuals’ capacity to secure decent work, which in turn fulfills fundamental human needs for survival, social connection, and self-determination [44, 45]. Blustein et al. have argued that a psychological perspective can revitalize the DWA by infusing a more specific focus on individual experiences and by reconnecting decent work to its social justice origins, particularly in relation to the rise of precarious work [46]. The development of the Decent Work Scale by Duffy et al. has enabled empirical measurement at the individual level, complementing the macro-level indicators developed by the ILO [47]. Pouyaud has further proposed a psychosocial approach to decent work that integrates subjective experience with structural determinants [48]. These psychological approaches to decent work resonate with CST’s emphasis on the subjective dimension of work articulated in *Laborem Exercens*, where Pope John Paul II argued that the value of work is determined primarily by the dignity of the person performing it [20]. A particularly urgent challenge to the DWA is the persistence of the informal economy, which according to ILO estimates encompasses approximately two billion workers globally, representing over 61% of total employment [49, 50]. Workers in the informal economy typically lack access to social protection, OHS, and fundamental labor rights (conditions that are diametrically opposed to the principles of decent work) [44, 45]. The ILO’s Recommendation No. 204 (2015) on the Transition from the Informal to the Formal Economy established for the first time an international normative framework to guide this transition, recognizing that most people enter the informal sector not by choice but out of a lack of opportunities in the formal economy [51]. From the perspective of CST, the condition of informal workers exemplifies the structural injustice that *Dilexi Te* denounces: economic systems that exclude the most vulnerable from the protections and dignity that work should confer [29].

### Occupational medicine as a pillar of decent work

Occupational Medicine (OM) is the medical specialty concerned with the prevention, diagnosis, and management of work-related diseases and injuries. Since 1950, the ILO and the World Health Organization (WHO) have maintained a common definition of occupational health, which aims to promote and maintain the highest degree of physical, mental, and social well-being of workers; to prevent departures from health caused by working conditions; to protect workers from risks adverse to health; and to adapt work to the capabilities of individual workers [6]. This definition was revised at the Twelfth Session of the Joint ILO/WHO Committee on Occupational Health in Geneva in 1995 [6]. The ILO Occupational Health Services Convention (No. 161, 1985) defines OHS as services with essentially preventive functions, responsible for advising employers, workers, and their representatives on establishing and maintaining a safe and healthy working environment [36]. The 1996 WHO Global Strategy on Occupational Health for All further emphasized the importance of a multidisciplinary approach and multisectoral collaboration in the provision of OHS [52]. The ILO’s Promotional Framework for Occupational Safety and Health Convention (No. 187, 2006) subsequently reinforced the systemic approach, calling for the continuous improvement of national OSH systems [53]. Globally, the scale of occupational disease and injury remains staggering. According to WHO and ILO joint estimates, nearly 2 million people die each year from exposure to occupational risk factors, and an additional 374 million non-fatal work-related injuries occur annually [54]. Work-related health problems result in an economic loss of 4–6% of gross domestic product (GDP) for most countries, and approximately 70% of workers worldwide lack insurance coverage for occupational diseases and injuries [55]. Chirico has further questioned whether GDP growth alone can serve as a valid indicator of decent work, arguing that health outcomes must be integrated into any meaningful assessment [56]. These figures underscore the urgency of integrating robust OHS into national decent work strategies. A global survey conducted by Rantanen, Lehtinen, and Valenti across International Commission on Occupational Health (ICOH) member countries revealed that, while the qualitative content and multidisciplinary nature of OHS generally correspond to international guidance, the coverage, comprehensiveness, and content of services remain largely incomplete due to a lack of infrastructure and a shortage of multiprofessional human resources [57]. This finding highlights the persistent gap between normative aspirations and practical realities, a gap that both CST and the DWA seek to close.

### The convergence of catholic social teaching and occupational health

A comparative analysis of CST and the ILO’s decent work framework reveals a striking convergence of principles. Before mapping this convergence in detail, however, it is necessary to provide a more explicit account of what “decent work” means and how it relates to CST. As formally defined by the ILO, decent work denotes productive employment that is carried out in conditions of freedom, equity, security, and human dignity [5]. Its four strategic pillars, employment creation, social protection, rights at work, and social dialogue, are complemented by a multidimensional measurement framework that encompasses 11 substantive elements, from adequate earnings and safe working conditions to work-family balance, social protection, and the broader economic and social context [40]. Crucially, the ILO framework is operative and governance-oriented: it identifies standards, indicators, and policy levers, and its authority derives from international labor law and the tripartite consensus of governments, employers, and workers’ organizations. CST, by contrast, grounds its vision of work in a theological anthropology: the dignity of the worker is not a policy construct but an ontological reality rooted in the imago Dei, and the norms that flow from it carry a moral weight that transcends legal or economic categories. This difference in grounding entails a difference in scope and emphasis. The ILO DWA is, by design, universal, secular, and measurable; its criteria must be applicable across diverse cultural and religious settings. CST brings an explicitly transcendent horizon, in which work is understood not only as a social right but as a vocation, a participation in God’s creative activity and a means of personal sanctification. The ILO framework, furthermore, does not directly address the theological meaning of rest, the spiritual dimensions of work, or the call to serve the common good as a matter of religious duty, elements that are constitutive of the CST vision. Conversely, while CST has consistently affirmed the rights of workers and called for structural reform, it has not developed the empirical measurement tools or the institutional governance mechanisms that the ILO has built up over more than a century of international norm-setting. A further distinction concerns the treatment of productivity. The ILO definition explicitly links decent work to productive employment, reflecting a concern for the sustainability of economic systems; CST, while not dismissing productivity, consistently subordinates it to the primacy of the person and the requirements of the common good, warning against any reduction of the worker to a factor of economic output. These distinctions do not undermine the convergence noted throughout this article, but they clarify it: the two frameworks are complementary rather than identical, each supplying what the other lacks, CST providing the anthropological depth and moral motivation that secular governance frameworks cannot generate from within themselves, and the ILO framework providing the institutional architecture, legal enforceability, and empirical rigor that ecclesial teaching alone cannot deliver. It is precisely this complementarity that makes their dialogue so valuable for the practice of OM. Both traditions affirm that work must respect human dignity and must not be reduced to a mere factor of production. Both recognize the right of workers to safe and healthy working conditions, to reasonable working hours, to rest, and to protection from hazards that endanger physical or mental health. Both emphasize the importance of social protection systems that provide security in the event of illness, injury, or unemployment. And both advocate for the active participation of workers in decisions that affect their well-being, whether through trade unions (as emphasized in CST) or through social dialogue (as emphasized in the DWA) [4, 7, 25]. What CST contributes to the discourse, beyond the normative framework of international labor law, is a robust philosophical and theological anthropology that grounds these principles in the inherent dignity of the human person, created in the image of God and “ut operaretur” (so that he might work) (cf. Genesis 2:15) [1, 2]. This grounding asks for a renewed reflection about the strict relationship between human dignity and human work, laying the foundations for a common reflection about the specific contents of decent work and on the future of human work too. It also provides a deeper source of moral motivation for the protection of workers’ health, one that transcends utilitarian calculations of cost and benefit and appeals instead to the intrinsic value of every human life. The role of OM, within this convergent framework, is therefore not merely technical but profoundly ethical. The occupational physician, in safeguarding the health of workers, participates in a mission that CST would recognize as a form of service to the common good, a concrete expression of the solidarity and justice that both the Church and international institutions call for [54]. From the perspective of *Dilexi Te*, the occupational health professional who serves workers in hazardous industries, in the informal economy, or in low-resource settings is engaged in precisely the kind of preferential care for the poor and vulnerable that the document commends [29]. As LaDou has critically observed, the gap between the promises of international organizations and the reality of occupational health on the ground remains a moral scandal that calls for renewed commitment from all stakeholders [38].

## Discussion

The historical trajectory from *Rerum Novarum* to *Dilexi Te* reveals a consistent and deepening commitment within CST to the protection of workers and the conditions of their labor. Pope Leo XIII’s denunciation of exploitative working conditions in the age of industrialization finds its contemporary echo in Leo XIV’s analysis of the structural causes of poverty and his call for a response that integrates moral conviction with scientific and technological innovation [11, 29]. The convergence between CST and the ILO DWA is neither coincidental nor merely rhetorical. Both frameworks emerged from a shared historical experience of witnessing the human cost of economic systems that prioritize profit over persons. The ILO’s founding in 1919, just 28 years after *Rerum Novarum*, was itself a response to the same social upheaval that had prompted Pope Leo XIII’s intervention [8]. The two traditions have developed in parallel, each enriching the other, and their convergence in the twenty-first century offers a powerful basis for advocacy and policy development in the field of occupational health [37]. However, significant challenges remain. Despite the progress achieved through the DWA, OHS remain inaccessible to the majority of workers worldwide, particularly in LMIC, in the informal economy, and among migrant and precarious workers [7, 55]. The rapid pace of technological change, including the advent of AI and automation, poses new risks to workers’ health and employment security that existing international standards have only begun to address; within CST, however, Pope Leo XIV’s *Magnifica Humanitas* (2026) now offers a sustained magisterial response, framing AI as the defining social question of the present era and calling for its governance to be oriented toward human dignity and the common good [33, 35]. It is worth considering, in greater detail, how CST might concretely help to address the specific pressures and barriers that practitioners of OM face in LMIC and other resource-constrained settings. Several barriers are particularly acute in these contexts: the absence or weakness of regulatory frameworks for OSH; chronic underfunding of OHS infrastructure; a severe shortage of trained occupational health professionals; the dominance of informal and precarious employment; cultural and linguistic barriers to health promotion; and the limited political leverage of workers’ organizations in the face of powerful economic interests. CST can contribute to addressing these barriers through at least four channels. First, at the institutional level, Catholic healthcare networks and Caritas organizations are among the largest providers of social and health services in low-income countries, and their explicit commitment to the principles articulated in *Dilexi Te* and *Magnifica Humanitas* could translate into concrete investments in workplace health programs, particularly in sectors, such as agriculture, artisanal mining, and domestic work, where formal OHS coverage is minimal. Second, the CST principle of subsidiarity, which holds that decisions should be made at the most appropriate local level while higher authorities provide enabling support, offers a governance rationale for decentralized and community-based OHS delivery models that are better adapted to local realities than top-down national programs. Third, the moral authority of local religious communities and leaders can be a powerful lever for raising awareness of occupational health rights among workers who may be unaware of or indifferent to abstract international standards, but who are responsive to appeals grounded in the dignity of the human person and the demands of justice articulated in their own religious tradition. Fourth, the CST emphasis on solidarity across national boundaries provides a theological foundation for the kind of South-South and North-South cooperation in OHS capacity-building that is essential to closing the global gap in occupational health coverage. These contributions are not merely theoretical. The Church’s engagement with labor issues in Latin America, Africa, and Southeast Asia, through trade union formation, legal aid for workers, and advocacy in international forums, demonstrates that CST principles can be operationalized in resource-constrained settings when backed by organized ecclesial action. The challenge for the occupational health community is to identify and strengthen these synergies, recognizing that in many LMIC contexts, Catholic institutions and CST-inspired civil society organizations may be among the most credible and effective partners available for the advancement of the DWA. Fletcher et al. have demonstrated that poor working conditions have cumulative negative effects on health, with disproportionate impacts on women, older workers, and racial minorities [58]. The integration of occupational health into primary healthcare systems, as recommended by the WHO, represents a promising avenue for expanding access, but requires sustained political will and investment [55]. Recent scholarship has further illuminated the nexus between decent work, health outcomes, and sustainable development. Schulte et al. have proposed a staging framework for expanding the role of OSH within the decent work paradigm, arguing that OSH professionals must move beyond traditional hazard-based approaches toward a more comprehensive engagement with the social determinants of work-related health [59]. Mariappanadar has examined the interactions between SDG 3 (Good Health and Well-being) and SDG 8 (Decent Work and Economic Growth), demonstrating that the health harm of work represents a critical yet often overlooked dimension of sustainability reporting [60]. A meta-analysis by Su and Chan has confirmed significant positive associations between perceived decent work and work engagement, motivation, and overall well-being, providing empirical support for the theoretical frameworks articulated by both the ILO and CST [61]. Burchell et al. have further contributed to this discourse by distinguishing between various methodological approaches to measuring employment quality, noting that definitions of decent work remain contested and politically sensitive [62]. Di Fabio and Maree have proposed a transdisciplinary interpretive lens that integrates philosophical, legal, economic, sociological, and psychological perspectives on decent work, an approach that aligns with CST’s own comprehensive vision of the human person [63]. These converging lines of research underscore the need for a truly interdisciplinary approach to decent work; one that integrates the ethical depth of CST, the normative rigor of international labor standards, and the empirical precision of occupational health science. From the perspective of CST, the challenge is also one of implementation. The principles articulated in papal documents must be translated into concrete institutional practices within Catholic healthcare systems, educational institutions, and advocacy organizations. The Compendium of the Social Doctrine of the Church emphasizes that these principles are understandable by all persons of good will and provide a basis for collaboration across confessional and cultural boundaries [1]. The occupational physician, in this context, serves as a crucial mediator between ethical principle and clinical practice, ensuring that the dignity of the worker is respected not only in theory but in the daily reality of the workplace.

### Beyond convenience: New perspectives in the age of digital transformations

It might be argued that the fulfillment of SDGs 3 and 8 in the age of technological transformations might require a specific attention to decent work strategies also because of mere economic convenience. Organizations and organizational infrastructures, in fact, will not be sustainable in the current scenarios. Although this might be true in pragmatic terms, we argue that a proper discussion about decent work in the light of the Catholic tradition might be able to open new scenarios and possible agendas for effective common actions. In the context of contemporary technological transformations, human work is increasingly framed not only in utilitarian terms but also increasingly within functionalist and efficiency-driven paradigms, where both human agents and artificial systems are understood primarily as decision-making structures aimed at optimizing outcomes. This perspective suggests that part of the OM agenda also has to take into account a deep anthropological crisis in the organizational and subjective perception of work and reflects a reductionist bias also in the criteria of personal satisfaction and success in a workplace. That is, current technocratic scenarios tend to exacerbate a reductionist and mechanistic epistemology of human work that tends to equate rationality with calculability and control, thereby narrowing the scope of human action to measurable performance. Such an approach fails to account for the intrinsically relational and meaningful nature of human work. A transition “from consensus to conviction” thus marks a crucial epistemic and ethical shift: rather than grounding social coordination in procedural agreement alone, it requires a shared understanding rooted in the dignity of the human person and in a fundamental sense of belonging to the human community, and in a shared commitment to serve and contribute to the common good. As highlighted in *Fratelli Tutti*, a just society cannot rely merely on externally imposed norms, but must be sustained by a “conviction […] that each human being is sacred and inviolable” (Francis, FT, n. 207) [28]. In this light, also the notion of “convenience” must be reinterpreted beyond utilitarian efficiency and reconnected to the common good, understood as the set of social conditions that enable both individuals and communities to flourish (Compendium of the Social Doctrine of the Church, n. 164). Such a reorientation is particularly urgent in the era of technological mediation of work, where the delegation of tasks to artificial systems risks eroding human responsibility and judgment. Human work itself, therefore, must be rethought as a practice that integrates technical competence with ethical discernment and relational responsibility, grounded in an anthropological vision that recognizes the unity, dignity, and social nature of the human person [64]. This reorientation finds explicit magisterial expression in Pope Leo XIV’s *Magnifica Humanitas*, which names the “technocratic paradigm” as a central danger of the digital age and warns against treating the human being as something to be optimized or surpassed, insisting instead that true progress is measured by the dignity of each person and the good of all peoples [35]. By calling believers and all people of goodwill to remain “profoundly human” amid the transformations wrought by AI, the encyclical supplies precisely the anthropological grounding that an OM attentive to the meaning of work, and not merely to its measurable performance, requires [35].

## Conclusions

This article has traced the development of Catholic Social Teaching on work and workers’ health from *Rerum Novarum* (1891) to *Dilexi Te* (2025) and *Magnifica Humanitas* (2026), demonstrating a consistent commitment to the dignity of labor, the protection of workers from exploitation and harm, and the promotion of conditions that support integral human development. The convergence between CST and the ILO’s decent work framework provides a compelling ethical and practical basis for advancing occupational health at the global level. The role of OM in this enterprise is both scientific and ethical. By safeguarding the physical, mental, and social well-being of workers, OM embodies the principles of justice, solidarity, and preferential concern for the vulnerable that are at the heart of both Catholic social thought and international labor standards. As Pope Leo XIV’s *Dilexi Te* reminds us, the faces of the poor and the suffering reveal the heart of Christ, and our response to their needs, including their occupational health needs, is a measure of our fidelity to the Gospel and to our common humanity [29]. Future research should explore the practical implementation of these principles within specific occupational settings, particularly in LMIC and among workers in the informal economy. The insights offered by the PWT and the growing body of empirical research on decent work measurement provide valuable tools for assessing the impact of both policy interventions and ethical frameworks on workers’ lived experiences [44, 47, 61]. Interdisciplinary dialogue between theologians, ethicists, occupational health professionals, and policymakers will be essential to ensure that the vision articulated by CST and the ILO DWA is translated into effective action for the protection and promotion of workers’ health worldwide [65]. In an age increasingly shaped by AI, the appeal of *Magnifica Humanitas* to remain “profoundly human” and to direct technological power toward the dignity of every person lends this task a renewed urgency and a fresh magisterial mandate [35].

## Data Availability

All data generated or analysed during this study are included in this published article. No additional datasets were generated.
